# Evaluating Ethnic Disparities in Restrictive Practices in Broadmoor High Secure Hospital

**DOI:** 10.1192/bjo.2025.10503

**Published:** 2025-06-20

**Authors:** Laura Gröger, Lauren Boniface, Susanna Martin, Saji Nabi

**Affiliations:** 1 Broadmoor Hospital, West London, United Kingdom; 2 National High Secure Consultants Forum, National, United Kingdom

## Abstract

**Aims:** Manual and mechanical restraint are restrictive practices that are applied as a last resort in a high secure psychiatric setting in order to manage risk to self, others and to deliver safe care. These interventions can have inherent risks to the physical and mental health of patients and staff. Previous studies have shown a discrepancy in the way patients from different ethnic backgrounds can experience restrictive practice in mental health care settings. This service evaluation aims to understand whether a patient’s ethnicity has an influence on the use of manual and mechanical restraint at Broadmoor High Secure Hospital by considering restraint variables alongside demographic and risk factors.

**Methods:** This quantitative study involved the retrospective data collection of all manual and mechanical restraints in the hospital between April 2023 to April 2024. Manual restraints included 63 patients and 354 incidents. Mechanical restraints included 12 patients and 70 incidents.

Demographic variables included patient ethnicity, length of admission, index offence and psychiatric diagnosis. Restraint variables included frequency, duration, type, reason for restraint and target of the incident.

**Results:** Inferential analysis showed no statistical difference between the ethnic distribution of the manually restrained patient population and the ethnic distribution of the whole hospital patient population.

Descriptive analysis found varied distributions of restrictive practices across ethnic groups. Further inferential statistics revealed a significant difference between ethnic groups for manual restraints due to self-harm. Correlational analysis revealed a significant positive relationship between length of admission and frequency of manual restraints across a one-year period.

**Conclusion:** This service evaluation explored the use of restraint practices among patients of differing ethnicities within Broadmoor High Secure Hospital, enabling clinical and research recommendations to be made. This project highlighted varied distributions in relation to how different ethnic groups experience manual and mechanical restraint. Future projects should include a dataset spanning over a larger number of years to enable more robust conclusions to be drawn on whether there are ethnic disparities in restrictive practices. Future projects should also involve qualitative data from patients and staff to better understand the complexities surrounding the treatment of differing ethnicities within mental health care settings. The authors of this service evaluation have already planned to look at the use of short term seclusion and long term segregation among differing patient ethnic groups within Broadmoor High Secure Hospital, to further understand this critical issue.

